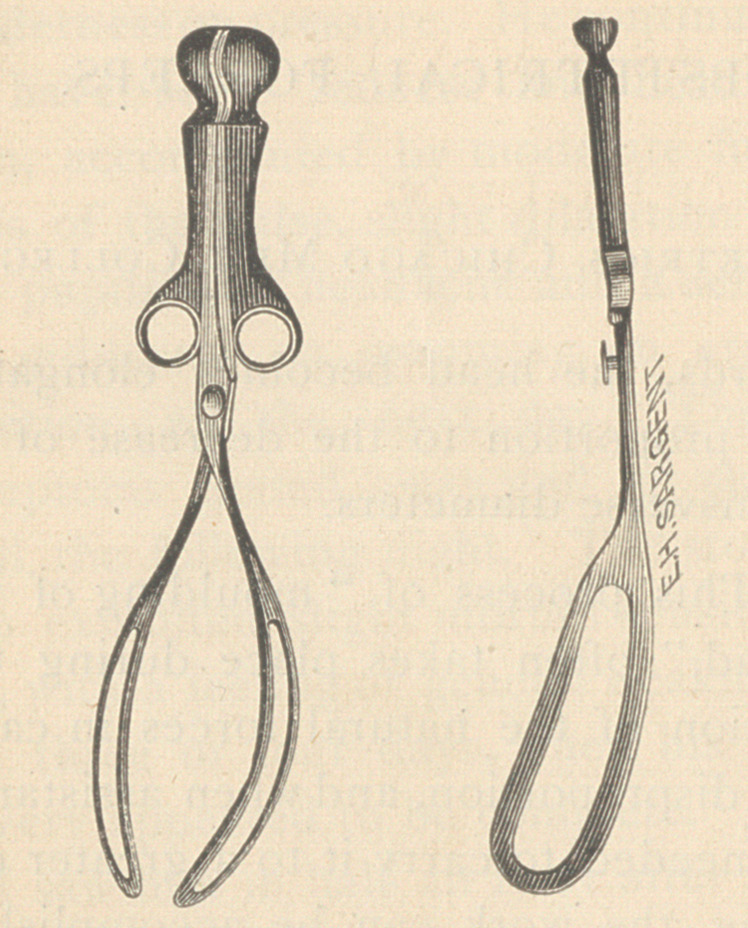# A Modification of the Obstetrical Forceps

**Published:** 1874-10-01

**Authors:** E. O. F. Roler

**Affiliations:** Prof. of Obstetrics, Chicago Med. College; 1105 Indiana av.


### A MODIFICATION OF THE OBSTETRICAL FORCEPS.

By E. O. F. Roler, M.D., Prof. of Obstetrics, Chicago Med. College.

IN obstetrical forceps of any pattern the modification may have reference either to the ease of introduction and adjustment to the head, or to the kinds of force employed in extraction, viz.: traction, leverage and compression.

This last kind of force can be made available only in instruments of considerable strength of blade and with long handles.

The circumstances admitting of its employment, however, occur but rarely. Compression is admissible only in the comparatively rare cases where traction combined with the leverage movement fails to secure the advance of the head. A resort to it always imperils the safety of the child, and the advantages to be gained by such a procedure are so uncertain that most obstetric authorities are averse to recommending it except as a destructive operation. And in order to prevent the possibility of injury in the hands of the unskilled, most forceps are provided either with "short handles," or they are of such moderate length that no great amount of compressing force can be applied in the act of extraction.

Experience of late years has demonstrated the fact that when the bi-parietal diameter is lessened by compression, a corresponding increase takes place in the long diameters of the head, the increase of the occipito-bregmatic being prevented by the resistance of the pelvic walls; in other words, the head becomes elongated in proportion to the decrease of its transverse diameters.

This process of "moulding of the head," often takes place during the action of the natural forces in cases of disproportion, and when assistance is needed to carry it to a greater degree, the work can be accomplished more safely by patient traction, and, if need be, aided by leverage, the *vis a fronte* reinforcing simply the *vis a tergo*.

While these facts are generally recognized in the application of the forceps, the varieties in use are, for the most part, of unnecessary strength and weight. The samples of German forceps especially which reach this country are notable for clumsiness in this respect. In the anxiety of their operators to avoid the dangers of compression the handles are made almost too short for convenient grasping, while the strength of the instrument is sufficient to admit of use for cephalotripsy. The same fault, though in a lesser degree, may be urged against most of the forceps in use among the profession in our own country.

Several years ago I had a pair of forceps made of the lightness indicated, with modifications of shape to suit my ideas of convenience in handling, and with but few exceptions have used them with great satisfaction in my cases of assisted labor.

The following cut will give a good idea of their shape.

A brief description is as follows: total length, 14 inches; length of handles, 6 inches, with a gradual pelvic curve of 2½ inches, beginning near the extremity of the shanks. The breadth of the cephalic portion averages 1½ inches, and has nearly the same measurement throughout, so that the fenestræ instead of being "kite-shaped," are nearly elliptical, being slightly larger at the outer extremity. When the instrument is closed the distance across the widest part is three inches, the points being separated three-fourths of an inch.

The branches fit loosely in a "button-lock," and the handles are supplied with rings. The total weight is about 13 ounces while that of ordinary forceps of approximate length varies from 18 to 21 ounces.

When applied, the right hand grasping the handles from beneath, with two fingers in the rings, can command the instrument in making traction with the most perfect ease, while the left can be used in assisting it, or, when needed, in supporting the distended perineum.

The advantages to be gained in forceps of such light construction and of the shape described, are:

1st. Their *lightness*, which adds greatly to the ease and readiness of introduction, and even adjustment, just as a delicate probe is carried to an obscure point in the tissues with more certainty and tact than a heavier one.

2d. The space within the cephalic portion so nearly corresponds to the bi-parietal diameter, averaging 3½ inches, that no dangerous compression would ensue on firm closure of the handles, allowing at least one-half the difference to be accounted for in the springing of the blades.

3d. The greater curvature of the blades, as compared with Hodges' and most other forceps, secures a firmer grasp upon the head, so that when properly applied slipping is practically impossible.

4th. They can be readily applied without disturbing the patient in the ordinary obstetric position on the back, and thus afford, with but little show of an operation, all the additional force required in the great majority of cases of instrumental labor.

I am under obligations to Mr. E. H. Sargent, instrument dealer in this city, for the finely executed cut accompanying this article, taken from his Catalogue of Instruments. The forceps may be found at his establishment, 785 Wabash avenue.

1105 Indiana av.

---

BELLADONNA IN GOITRE.—Dr. R. T. Smith reports two cases of exophthalmic goitre, which were relieved and almost cured by the free administration of tincture of belladonna. He considers it possible that relief was given primarily through the heart, the drug acting sedatively hereon.—*Phil. Med. Times.*

## Figures and Tables

**Figure f1:**